# Ser649 and Ser650 Are the Major Determinants of Protein Kinase A-Mediated Activation of Human Hormone-Sensitive Lipase against Lipid Substrates

**DOI:** 10.1371/journal.pone.0003756

**Published:** 2008-11-19

**Authors:** Christian Krintel, Peter Osmark, Martin R. Larsen, Svante Resjö, Derek T. Logan, Cecilia Holm

**Affiliations:** 1 Division of Diabetes, Metabolism and Endocrinology, Department of Experimental Medical Science, Lund University, Lund, Sweden; 2 Department of Molecular Biophysics, Lund University, Lund, Sweden; 3 Department of Biochemistry and Molecular Biology, University of Southern Denmark, Odense, Denmark; The Research Institute for Children at Children's Hospital New Orleans, United States of America

## Abstract

**Background:**

Hormone-sensitive lipase (HSL) is a key enzyme in the mobilization of fatty acids from stored triacylglycerols. Its activity is regulated by reversible protein phosphorylation. In rat HSL Ser563, Ser659 and Ser660 have been shown to be phosphorylated by protein kinase A (PKA) *in vitro* as well as *in vivo*.

**Methodology/Principal Findings:**

In this study we employed site-directed mutagenesis, *in vitro* phosphorylation and mass spectrometry to show that *in vitro* phosphorylation of human HSL by PKA occurs primarily on Ser649 and Ser650 (Ser659 and Ser660 in rat HSL). The wild type enzyme and four mutants were expressed in C-terminally His-tagged form in Sf9 insect cells and purified to homogeneity. HSL variants in which Ser552 and/or Ser554 were mutated to Ala or Glu retained both lipolytic and non-lipolytic activity and were phosphorylated by PKA and activated to a similar extent as the wild type enzyme. ^32^P-labeling studies revealed that the bulk of the phosphorylation was on the Ser649/Ser650 site, with only a minor phosphorylation of Ser552 and Ser554. MS/MS analysis demonstrated that the peptide containing Ser649 and Ser650 was primarily phosphorylated on Ser650. The mutant lacking all four serines had severely reduced lipolytic activity, but a lesser reduction in non-lipolytic activity, had S_0.5_ values for p-nitrophenol butyrate and triolein comparable to those of wild type HSL and was not phosphorylated by PKA. PKA phosphorylation of the wild type enzyme resulted in an increase in both the maximum turnover and S_0,5_ using the TO substrate.

**Conclusions:**

Our results demonstrate that PKA activates human HSL against lipid substrates *in vitro* primarily through phosphorylation of Ser649 and Ser650. In addition the results suggest that Ser649 and Ser650 are located in the vicinity of a lipid binding region and that PKA phosphorylation controls the accessibility of this region.

## Introduction

Fatty acids mobilized from stored triacylglycerols are a major energy source in humans. Mobilization occurs through the consecutive action of three lipases: the recently discovered adipose triglyceride lipase, hormone-sensitive lipase (HSL) and monoacylglycerol lipase. Whereas adipose triglyceride lipase and monoacylglycerol lipase appear to be quite specific for triacylglycerols and monoacylglycerols, respectively, HSL exhibits very broad substrate specificity, hydrolyzing a wide range of substrates including triacylglycerol, diacylglycerol, monoacylglycerol, cholesteryl, retinyl, lipoidal and water-soluble esters. Accordingly, HSL is expressed in several tissues in addition to white adipose tissue, e.g. brown adipose tissue, skeletal muscle, steroidogenic tissues, intestine, pancreatic β-cells and macrophages. Its role in each of these tissues remains to be determined, but it seems clear that, in addition to its role in fatty acid mobilization, HSL plays an important role in lipid signalling events of importance for e.g. spermatogenesis, adipogenesis and insulin secretion. Thus, HSL is an important target for preventive and interventive actions in the area of obesity and diabetes [1].

A major feature of HSL is its regulation by reversible protein phosphorylation. Early work by Belfrage and others showed that HSL was phosphorylated at two serines in what was later designated the regulatory module of the enzyme [2], [3], [4], [5], [6]. One site was phosphorylated under basal conditions (the basal site) and another was phosphorylated by protein kinase A (PKA) upon stimulation of lipolysis (the regulatory site). In the rat enzyme these phosphorylation sites correspond to Ser563 (regulatory site) and Ser565 (basal site). The basal site has been reported to be phosphorylated *in vitro* by several kinases, with AMP-activated protein kinase (AMPK) as the most likely candidate *in vivo* [3], [4]. It has been suggested that phosphorylation of these two sites is mutually exclusive due to steric hindrance. *In vitro* PKA phosphorylation of recombinant HSL has been shown to increase the activity of the enzyme against triolein by 100% for the rat enzyme and by somewhat less for the human enzyme [7], [8].

During the last decade the phosphorylation events of rat HSL have proven to be more complicated than originally reported, with the demonstration that Ser659 and Ser660, in addition to Ser563, are phosphorylated by PKA *in vitro* as well as in response to lipolytic stimulation of rat adiopocytes. It was also found that Ser659 and Ser660 were the major activity controlling sites *in vitro*, whereas mutation of Ser563 has shown to be without effect with regard to both *in vitro* activation by PKA [9] and translocation in response to lipolytic stimulation of adipocytes [10]. In contrast, another study reported that mutating Ser563 to Ala abolished all HSL activity *in vitro,* indicating an important role for this residue in the enzymatic activity of HSL [11]. The phosphorylation sites of rat HSL are conserved in human HSL: Ser563, Ser565, Ser659 and Ser660 in the rat enzyme correspond to Ser552, Ser554, Ser649 and Ser650 in the human enzyme [8]. However, to date only a few studies have addressed the phosphorylation events in human HSL. These have shown that human HSL is activated to a lesser extent than rat HSL *in vitro* and have furthermore suggested an important role for Ser650 in governing the activation *in vivo* [8], [12], [13].

In this study we used site directed mutagenesis, *in vitro* phosphorylation, titanium dioxide phosphopeptide enrichment and mass spectrometry (MS) in combination with activity studies to show that Ser649/Ser650 is the major determinant of activation of human HSL by PKA *in vitro* and that phosphorylation alters the kinetic behaviour of the enzyme with an increase in both the maximum turnover rate and the S_0.5_ towards the trioleoin substrate. Furthermore, we demonstrate that Ser649 and Ser650 are major determinants of the lipolytic activity but are of lesser importance for the non-lipolytic activity of the non-phosphorylated enzyme.

## Materials and Methods

### Construction of vectors

The previously described cDNA clone of human HSL [8] was modified using a standard PCR-based mutagenesis protocol, cloned into the pVL1393 shuttle vector from PharMingen (BD Biosciences) and sequenced. Recombinant baculovirus was generated using the Baculo-Gold kit (PharMingen) according to the manufacturer's protocol. Single virus particles were isolated from the inoculum by plaque assay followed by the generation of high titre virus stocks. The titres of these stocks were determined by plaque assay. In total five variants of human HSL were generated (listed in **Table 1**). Common to all variants was that a proline residue followed by eight histidines was added to the C-terminus to aid purification.

**Table 1:** Recombinant HSL variants

Variant	Mutation
WT	None
SVA-SS	Ser 554 to Ala
SVE-SS	Ser 554 to Glu
AVA-SS	Ser 552 and Ser 554 to Ala
AVA-AA	Ser 552 and Ser 554 to Glu

### Expression and purification of HSL variants

Sf9 insect cells were grown at 27°C in suspension cultures (160 rpm) in Sf-900 medium supplemented with 4% heat inactivated foetal calf serum and 1% penicillin/streptomycin (all from Gibco). For expression of HSL, cell cultures (2×10^6^ cells/mL) were infected at a multiplicity of infection of 10 followed by a 72 h expression period. Cells were harvested by centrifugation and resuspended in five pellet volumes of lysis buffer (20 mM Tris-HCl pH 8.0, 1 mM DTT, 1 mM EDTA, 1% C_13_E_12_, 10% glycerol).

The cell suspension was gently sonicated and centrifuged for 45 min at 4°C and 50000 g. The supernatant fraction was filtered through a 0.22 µm filter and loaded onto a Q-Sepharose Fast Flow anion exchange column (GE Healthcare). The column was washed with ten volumes of 50 mM NaCl, 20 mM Tris-HCl pH 8.0, 1 mM DTT, 1 mM EDTA, 0.01% C_8_E_4_, 10% glycerol, then eluted with approximately two column volumes of 1 M NaCl, 20 mM Tris-HCl pH 8.0, 0.1 mM DTT, 0.01% C_8_E_4_, 10% glycerol.

Protein eluted from the Q-Sepharose column was loaded directly onto a nickel affinity chromatography column (Ni-NTA Superflow; Qiagen), washed with ten volumes of 18 mM imidazole, 300 mM NaCl, 50 mM Tris-HCl pH 8.0, 0.1 mM DTT, 1% Triton X100, 10% glycerol and 15 volumes of 5 mM imidazole, 300 mM NaCl, 50 mM Tris-HCl pH 8.0, 0.1 mM DTT, 0.01% C_8_E_4_, 10% glycerol and eluted with a stepwise gradient towards 250 mM imidazole, 300 mM NaCl, 50 mM Tris-HCl pH 8.0, 1 mM DTT, 0.01% C_8_E_4_, 10% glycerol. The eluted protein was then dialysed overnight against 50 mM Tris-HCl pH 8.0, 300 mM NaCl, 1 mM DTT, 0.01% C_8_E_4_ and 10% glycerol and stored at –80°C. Protein amounts were measured by the 2D Quant method (GE Healthcare) and the Bradford method [14]. The latter underestimated HSL content by a factor of 1.5 relative to the 2D Quant method.

### Tryptophan fluorescence measurements

Eight µg of purified protein was excited at 296 nm in 1 ml of 50 mM Tris-HCl pH 8.0, 1 mM DTT, 0.01% C_8_E_4_ and 10% glycerol and emission was scanned from 300 nm to 400 nm in a Fluoromax-2 fluorometer.

### Detection of phosphorylation of HSL in insect cells

Tissue culture flasks were supplemented with 3×10^6^ Sf9 insect cells in 5 ml Sf-900 medium at 27°C. After cell adhesion the medium was removed and cells were infected at a MOI of 10. As a negative control cells were infected with the BaculoGold kit control virus. After infection for 1 h the inoculum was replaced by 4 ml fresh medium and incubated at 27°C. After approximately 60 h the medium was replaced with 2 ml of fresh medium containing 0.1 mCi/mL ^32^PO_4_^3−^ (GE Healthcare) and incubated for an additional hour. Medium was removed and cells were gently rinsed twice with 4 ml of medium before they were scraped in 1 ml of 300 mM NaAc, 20 mM Tris-HAc pH 8.0, 5% glycerol, 0.5 mM DTT, 10 mM imidazole and 1% C_8_E_4_. Cell suspensions were transferred to Eppendorf tubes and placed at –20°C for 3 h followed by thawing on ice. After thawing the lysed cells were centrifuged for 20 min at 20000 g and 4°C. The supernatants were transferred to fresh tubes, to which 300 µl of Ni-NTA agarose were added and incubated at 4°C for 1 h, followed by mild centrifugation and removal of the supernatant. The gel was then washed twice with 1 ml of 300 mM NaAc, 20 mM Tris-HAc pH 8.0, 5% glycerol, 0.5 mM DTT, 10 mM imidazole and 0.01% C_8_E_4_. The protein was eluted by resuspending the gel in of 150 µl of elution buffer consisting of 300 mM NaAc, 20 mM Tris-HAc pH 8.0, 5% glycerol, 1 mM DTT, 250 mM imidazole and 0.01% C_8_E_4_, followed by mild centrifugation and collection of the supernatant.

The eluted samples were then dialyzed overnight against 20 mM Tris-HAc pH 8.0, 10% glycerol, 1 mM DTT and 0.01% C_8_E_4_. The dialyzed samples were analyzed by SDS-PAGE, stained with Coomassie Blue, scanned and slab dried. ^32^P-labeled HSL was detected by digital imaging on a Fujix BAS-2000 (Fuji). Image-Quant TL software (GE Healthcare) was used for quantification of signals on gels.

### Dephosphorylation and PKA phosphorylation of HSL

Six µg of HSL were dephosphorylated in 100 µl volumes containing 80 µl of the above dialysis buffer, 10 U (1 U/µl) of calf intestinal phosphatase, 10 µl of 1 mM Tris-HCl pH 8.0, 200 mM MgCl_2_ supplemented with a cocktail of protease inhibitors (Roche Complete). The dephosphorylation reaction was performed for 1 h at RT. The phosphatase was then inactivated by the addition of 2 µl of 500 mM sodium orthovanadate. The subsequent PKA phosphorylation was performed by mixing 50 µl of the dephosphorylation reaction mixture with 7 µl of 10x PKA buffer, 15 µl 10 mM ATP and 25 U PKA (New England Biolabs). In control reactions PKA and ATP were omitted. For the detection of incorporation of phosphate by PKA 3 µl [γ-P^32^]ATP (10 mC_i_/mmol) was added to the phosphorylation reaction mixture and aliquots were taken after 5, 15 and 30 min of incubation and quenched by the addition of Laemmli buffer [15]. Samples were analyzed by SDS-PAGE, stained with Coomassie, scanned and slab dried. ^32^P-labeled HSL was detected as described above. For quantification of incorporated phosphate HSL, bands were excised from the gel, placed in scintillation vials containing 10 ml of scintillation liquid and quantified on a scintillation counter (Wallac 1414 liquid scintillation counter, Perkin Elmer). The original reactions were included as standards.

### Mass spectrometry analysis of phosphopeptides

The proteins were subjected to SDS-PAGE and protein bands where excised from the gel and dried by lyophilisation. The excised gel plug was washed in digestion buffer (50 mM NH_4_HCO_3,_ pH 7.8/acetonitrile (60/40)) and dried by vacuum centrifugation. Modified trypsin (8 ng/µl) dissolved in 50 mM NH_4_HCO_3,_ pH 7.8 was added to the dry gel pieces and they were incubated on ice for 1 h. After removing the supernatant additional digestion buffer was added and the digestion was continued at 37°C for 4–12 h.

The purification of phosphorylated peptides was performed using titanium dioxide (TiO_2_) chromatography, essentially as described previously [16], [17]. An aliquot of the peptides derived from the in-gel digest (5 µl, corresponding to 20%) was mixed with 35 µl TiO_2_ chromatography loading buffer (80% acetonitrile/5% TFA/1 M glycolic acid [17]. The solution was applied to a TiO_2_ micro-column and the liquid was gently pressed through the column using a fitted 1 ml plastic syringe. The column was washed with 10 µl loading buffer and 20 µl 80% acetonitrile/1% TFA. The bound phosphopeptides were eluted using ammonia water pH 11.3 (10 µl ammonia (25%) in 490 µl UHQ water).

The purified phosphopeptides were lyophilized prior to nano-liquid chromatography ESI-MSMS or further desalted on reversed-phase micro-columns prior to MALDI MS analysis as described previously [18].

### MALDI mass spectrometry

MALDI MS was performed on a Bruker Ultraflex instrument (Bruker Daltonics). All spectra were obtained in positive reflector ion mode. The matrix used was 2.5-dehydroxybenzoic acid (DHB) (20 mg/ml) in 50% acetonitrile, 0.1% TFA, 1% phosphoric acid [19]. The spectra were processed using the flexAnalysis software (Bruker Daltonics).

### Nano-liquid chromatography tandem mass spectrometry (nano-LC-MS)

The nano-LC-MS experiments were performed using an Orbitrap XL mass spectrometer (Thermo Electron). The sample was applied to an EASY nano-LC system (Proxeon Biosystems, Odense, Denmark). The peptides were concentrated on a 1.0 cm pre-column (75 µm inner diameter, 360 µm outer diameter, ReproSil–Pur C18 AQ 3 µm (Dr. Maisch, Germany)). The peptides were eluted from the pre-column using a gradient from 100% phase A (0.1% formic acid aqueous solution) to 40% phase B (0.1% formic acid, 80%acetonitrile) in 100 min at 200 nl/min directly on an 8 cm analytical column (50 µm inner diameter, 360 µm outer diameter, ReproSil–Pur C18 AQ 3 µm). Peptides were analyzed using a data dependent setup where one MS run was performed in the Orbitrap at a resolution of 60000 FWHM and five MSMS runs were performed in the linear ion trap. Each ion selected for MSMS was fragmented using multi-stage fragmentation for phosphopeptides [20] where the losses of phosphoric acid from the phosphopeptides were automatically selected for another round of fragmentation. The resulting fragment ion spectrum contains product ions from both the precursor and the neutral loss product, providing more information.

### Database searching

The fragment ion spectra were processed (smoothing, background subtraction and centroiding) using the program DTASuperCharge (http://msquant.sourceforge.net/). The processed files were subsequently searched against the human HSL protein sequence using an in-house Mascot server (version 2.1) (Matrix Science Ltd., London, UK). The search was performed choosing trypsin as enzyme. Oxidation (M), N-Acetyl (Protein), Phospho (STY) and IntactPhospho (STY) were chosen as variable modification. The data were searched with a peptide mass tolerance of ±5 ppm and a fragment mass tolerance of ±0.6 Da. A maximum of one missed cleavage was allowed. Phosphopeptides were manually evaluated.

### HSL activity assays

HSL lipolytic activity was measured against phospholipid-stabilized emulsions of the diacylglycerol analogue 1(3)-mono-[^3^H]oleoyl-2-O-mono-oleylglycerol (MOME), tri[^3^H]oleoylglycerol (TO) and cholesteryl [^14^C]oleate (CO). Briefly, labeled and non-labeled lipid substrates and phosphatidylcholine/phosphatidylinositol (3∶1) in cyclohexane solutions were dried under at stream of N_2_ followed by emulsification by sonication and addition of 2% BSA (MOME assay) or 5% BSA (TO and CO assays). Enzymes were diluted to a suitable concentration in 100 µl of 20 mM potassium phosphate, pH 7.0, 1 mM EDTA, 1 mM DTT and 0.02% BSA and 100 µl of the emulsified substrate was added and mixed. Reactions were typically incubated for a period of 30 min at 37°C before the reaction was quenched by the addition of 3.25 ml of methanol/chloroform/heptane (10∶9∶7) and 1.1 ml of 0.1 M potassium carbonate, 0.1 M boric acid pH 10.5. Samples were then vortexed and centrifuged and the content of released fatty acids in the upper phase was determined by scintillation counting. For all assays we confirmed that the reaction velocity was constant during the 30 min incubation period. The activity against the soluble para-nitrophenylbutyrate (pNPB) substrate was measured in a 1 ml reaction volume by following the development of absorbance at 400 nm [21], [22], [23], [24]. In saturation kinetics experiments activities towards sequentially diluted substrates were determined as described above. Substrates for TO saturation curve measurements were prepared by mixing the substrate emulsion with a solution prepared in exactly the same way but with no added TO. One unit of enzyme activity is equivalent to 1 µmol of fatty acids released/min at 37°C. Saturation curves were analyzed by non-linear regression in the GraphPad Prism 5.0 for Mac software package.

## Results

### Expression and purification of human HSL variants

**Figure 1:**
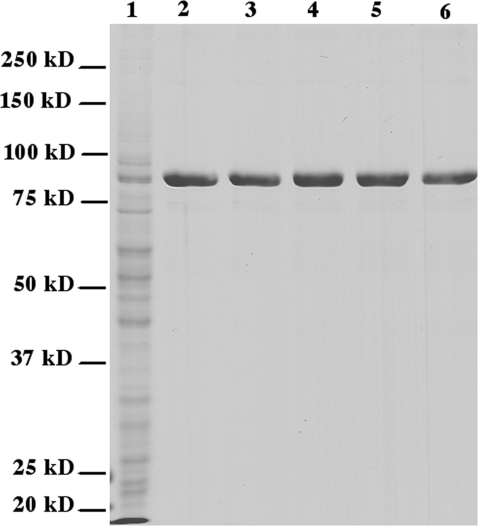
Expression and purification of HSL variants. Lane 1: soluble protein from Sf9 cells containing the wild type enzyme; Lane 2: purified wild type enzyme; Lane 3: purified SVA-SS variant; Lane 4: purified SVE-SS variant; Lane 5: purified AVA-SS variant; Lane 6: purified AVA-AA variant.

**Figure 2:**
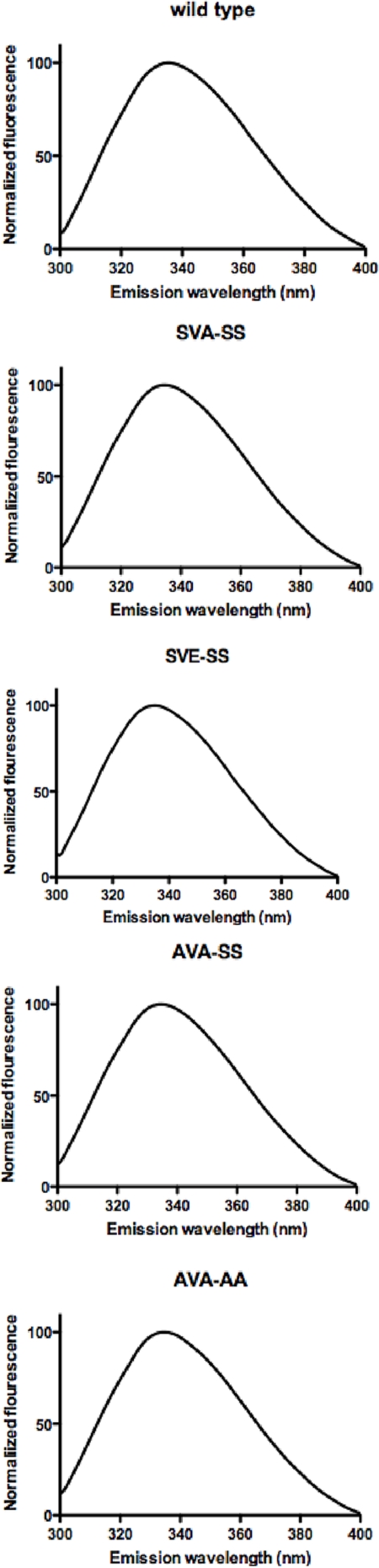
Normalized Trp fluorescence spectra of wild type, SVA-SS, SVE-SS, AVA-SS and AVA-AA variants of human HSL. Proteins were excited at 296 nm and emission was measured between 300 and 400 nm. Spectra were smoothened and normalized in GraphPad Prism 5.0 for Mac. The wavelength of the emission peaks is 335 nm for all five variants.

Five C-terminally His-tagged human HSL variants were successfully cloned and expressed in Sf9 insect cells using the baculovirus/insect cell expression system. These comprise a His-tagged wild type enzyme and variants in which Ser554 is mutated into alanine (SVA-SS), in which Ser554 is mutated to Glu (SVE-SS), in which both Ser552 and Ser554 are mutated to alanine (AVA-SS) and a quadruple mutant in which Ser552, Ser554, Ser649 and Ser650 are all mutated to Ala (AVA-AA; **Table 1**). All protein variants were purified by anion exchange chromatography followed by nickel affinity chromatography and dialysis. The purity of the protein was at least 95% as estimated by SDS-PAGE (**Figure 1**). Western blot analysis as well as MALDI MS analysis of tryptic digests confirmed the identity of the purified proteins (data not shown). The yield of pure protein was 2–5 mg per litre of insect cell culture. The specific activities of the purified wild type enzyme against TO, MOME, CO and pNPB were 1.2 U/mg, 23 U/mg, 2.3 U/mg, and 50 U/mg, respectively. Fluorescence emission spectra between 300 nm and 400 nm were recorded after excitation at 296 nm. All variants had a maximum emission wavelength of 335 nm (**Figure 2**).

### Background phosphorylation in insect cells

**Figure 3:**
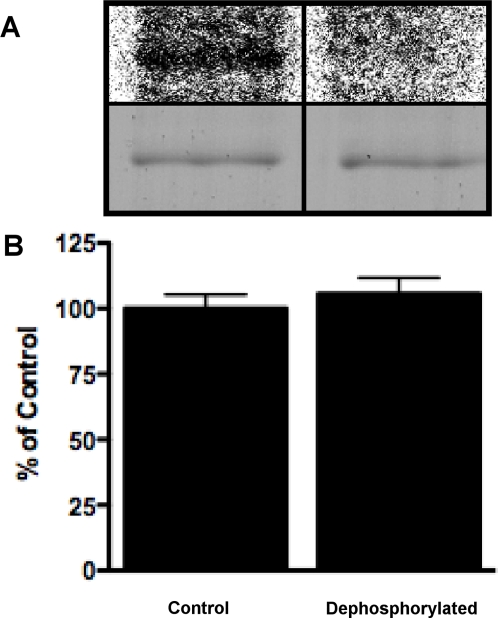
Phosphorylation status of HSL purified from Sf9 cells. The wild type enzyme was expressed in the presence of ^32^P, purified and dephosphorylated by calf intestinal phosphatase. The samples were analyzed by autoradiography (A; upper panel), by SDS-PAGE and Coomassie blue staining (A; lower panel) and by enzymatic assay using TO as substrate (B).

Incubation of cells with ^32^PO_4_^3−^ showed that wild type HSL is phosphorylated in Sf9 cells and that this phosphorylation can be removed by calf intestinal phosphatase (**Figure 3A**). Dephosphorylation of the wild type protein had no effect on triolein hydrolysis (**Figure 3B**). However, in order to exclude any differences in background phosphorylation between enzyme preparations a dephosphorylation step was included before activation experiments. Background phosphorylation was also present in the AVA-SS variant but could not be detected in the AVA-AA variant (not shown). MALDI MS analysis revealed phosphopeptides including Ser649 and Ser650 (not shown).

### Effects of point mutations on the activity of HSL

**Figure 4:**
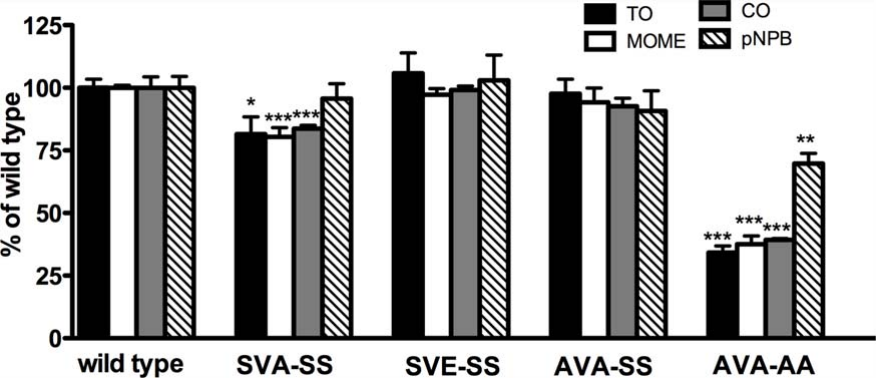
Activities of purified human HSL enzymes. Enzymes were assayed for their activity against TO, MOME and CO under saturated substrate concentrations. The activity against pNPB was performed with 1 mM pNPB. Data represent the means±standard error of nine assays. *P<0.05; *** P<0.0005, one way ANOVA.

Mutating Ser554 to alanine (the SVA-SS variant) resulted in a slight decrease in lipolytic activity. Mimicking the charge effect of an anti-lipolytic phosphorylation of the basal site by mutating Ser554 to glutamic acid (the SVE-SS variant) resulted in similar to wild type activity. Simultaneous mutation of both Ser552 and Ser554 to alanine (the AVA-SS variant) had no effect on the activity. However, mutating all four serines, including Ser649 and Ser650 (the AVA-AA variant) decreased the activity of human HSL against TO, MOME and CO substrates by 67%, 63% and 60%, respectively. The activity against pNPB also decreased, but only by 30% compared to wild type HSL (**Figure 4**).

### Kinetic behavior of the wild type enzyme and the AVA-AA mutant

**Figure 5:**
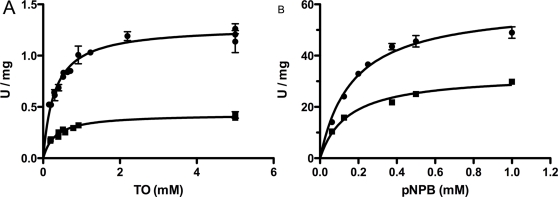
Saturation curves of the wild type enzyme (•) and the AVA-AA mutant (▪) in the TO assay (A) and the pNPB assay (B). Maximum turnover values were 1.3 U/mg and 0.42 U/mg for wild type and the AVA-AA mutant, respectively in the TO assay. The maximal turnover rates were 60 U/mg and 33 U/mg for wild type and the AVA-AA mutant respectively in the pNPB assay. The S_0.5_ values were 0.30 mM and 0.32 mM for the wild type enzyme and the AVA-AA mutant, respectively in the TO assay. The S_0.5_ values were 0.17 mM and 0.18 mM for the wild type enzyme and the AVA-AA mutant respectively in the pNPB assay (see also Table 2).

Both the WT enzyme and the AVA-AA mutant had Michaelis-Menten-like saturation curves when assayed against increasing concentrations of TO and pPNB (**Figure 5**). However, concepts and methods of enzymology, such as Michaelis-Menten kinetics, used to describe the kinetic behavior of enzymes acting in homogenous milieus do no apply to lipases acting in heterogenous reactions systems with two or more phases. For this reason we have used the terms maximum turnover rate and S_0.5_, corresponding to the substrate concentration leading to half of the maximum turnover rate, to describe the kinetic behavior of the different HSL enzymes. The S_0.5_ against TO was 0.30±0.023 mM for the wild type enzyme and 0.32±0.035 mM for the AVA-AA mutant. The maximum turnover rate for TO was 1.3±0.028 U/mg for wild type and 0.42±0.015 U/mg for AVA-AA. The S_0.5_ value for pNPB was 0.17±0.018 mM for wild type and 0.18±0.017 mM for AVA-AA. The maximal turnover rate for pNPB was 60±2.2 U/mg for wild type and 33±1.1 U/mg for AVA-AA (**Figure 5** **and** **Table 2**).

**Table 2:** Properties of purified wild type human HSL and the AVA-AA mutant

	Wild type	AVA-AA	Phosphorylated wild type
Maximum turnover in TO assay	1.3±0.028	0.42±0.015	2.3±0.11
S_0.5_ in TO assay	0.30±0.023	0.32±0.035	0.73±0.13
Maximum turnover in pNPB assay	60±2.2	33±1.1	
S_0.5_ in pNPB assay	0.17±0.018	0.18±0.017	

Units for turnover and S_0.5_ are U/mg and mM, respectively. Values are listed with ±SEM.

### *In vitro* phosphorylation by PKA

**Figure 6:**
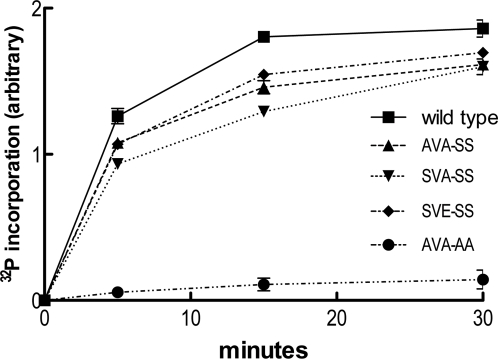
PKA phosphorylation of HSL variants. Purified HSL variants were dephosphorylated for 1 h with calf intestinal phosphatase and then rephosphorylated by PKA in the presence of ^32^P-labeled ATP. Reactions were subjected to SDS-PAGE and the amount of incorporated phosphate was determined by scintillation counting of gel bands and calculation from standards made from the original reactions.

Following inhibition of the phosphatase by 10 mM sodium orthovanadate, dephosphorylated HSL was phosphorylated by PKA. Phosphorylation reached its maximum after 30 to 45 min and the stoichiometry was determined to be 0.2 mol phosphate per mol protein for wild type HSL. The SVA-SS and SVE-SS variants in which Ser554 was replaced by alanine and glutamic acid (the latter to mimic an anti-lipolytic phosphorylation), respectively, and the AVA-SS variant in which both Ser552 and Ser554 had been substituted for alanine, were phosphorylated to a similar degree as wild type HSL. However, the AVA-AA mutant, where also Ser649 and Ser650 have been replaced by alanine, was poorly phosphorylated (**Figure 6**).

**Figure 7:**
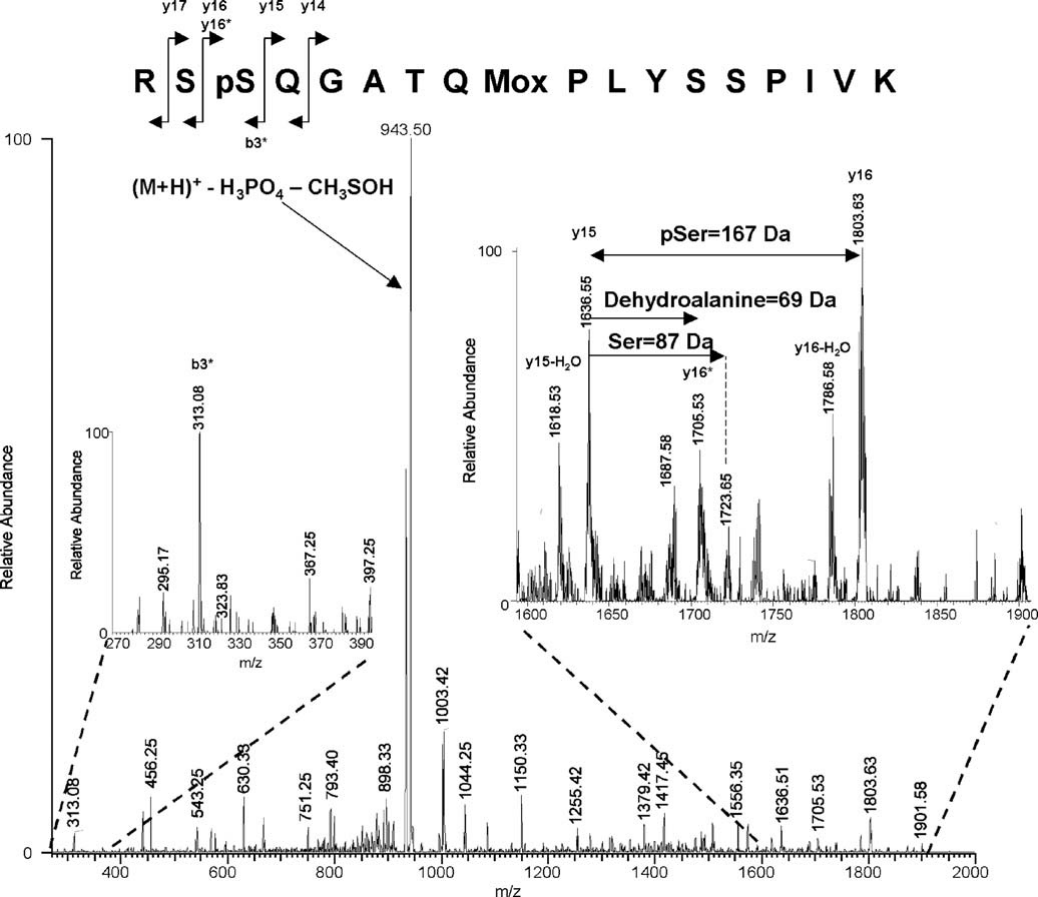
MS/MS spectrum of the mono-phosphorylated RSSQGATQMPLYSSPIVK peptide comprising both Ser649 and Ser650. Human HSL phosphorylated by PKA was digested with trypsin and phosphopeptides were isolated using TiO_2_. The isolated peptides were subjected to MS analysis as described in the experimental procedures section. Shown is the MS/MS spectrum of a doubly charged precursor ion (m/z = 1024.4 Da) corresponding to the peptide RSSQGATQMPLYSSPIVK (with Q4 deamidated, M9 oxidized and either S2 or S3 phosphorylated). The 943.50 Da peak corresponds to the selected parent ion with the loss of phosphate and methanesulfenic acid. From the y ion series (for instance y12 at 1379.42 Da and y15 at 1636.51 Da), it can clearly be seen that T7, S13 and S14 are not phosphorylated. The large peak at 1803.63 Da corresponds to the y16 ion from a peptide phosphorylated on S3 and would not be present if S2 was the only phosphorylation site. The smaller peak at 1723.65 Da, on the other hand, corresponds to the y16 ion containing no phosphoserine/threonine at all. The presence of both these peaks indicates that the parent ion consists of a mixture of tryptic peptides phosphorylated on S2 or S3. However, the peak at 1803.63 Da is derived from a fragment containing phosphate while the peak at 1723.65 Da is derived from the corresponding unphosphorylated fragment. All other things being equal, an unphosphorylated fragment would be expected to ionize better and give a stronger signal than the corresponding phosphorylated fragment. Thus, the observation that the peak at 1803.63 Da is so much larger than the peak at 1723.65 Da strongly indicates that the degree of phosphorylation of S3 is much higher than that of S2.

MS analysis of TiO_2_-prepared tryptic peptides of the phosphorylated enzymes detected phosphopeptides in all five enzymes. For the wild type, SVA-SS, SVE-SS and AVA-SS enzymes phosphorylation was found at the Ser649/Ser650 site. MS/MS analysis of the respective phosphopeptide suggested that the bulk of the Ser649/Ser650 site phosphate groups was present on Ser650 (**Figure 7**). For the wild type enzyme phosphorylation was also present on a peptide containing Ser552 and Ser554. For the SVA-SS mutant phosphorylation was found on Ser552, whereas no phosphorylation was detected on this site in the SVE-SS variant. Additionally, phosphorylation of Thr512 was detected in all of the five enzymes (**Table 3**).

**Table 3:** Phosphorylated peptides in PKA-treated HSL variants

Position	Peptide sequence	Found in variant
510–517	RDpTALLLR	All
552–571	p[SVS]EAALAQPQGPLGTDSLK	WT
648–665	RpSSQGATQMPLYSSPIVK	WT, SVA-SS, SVE-SS and AVA-SS
648–665	RpSSQGATQMPLYSSPIVK	WT, SVA-SS, SVE-SS and AVA-SS
552–571	pSVAEAALAQPQGPLGTDSLK	SVA-SS

### Activation of HSL variants by PKA phosphorylation

**Figure 8:**
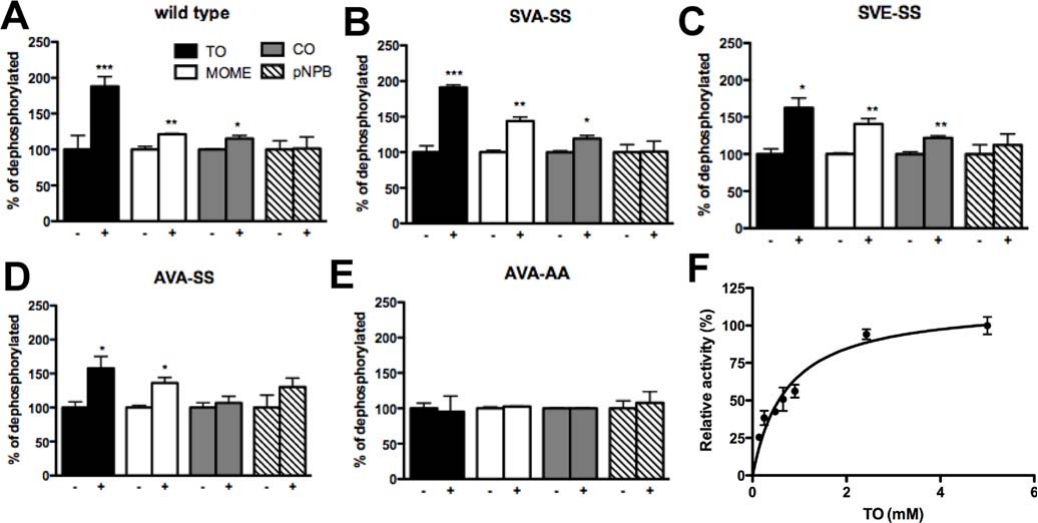
Activities of dephosphorylated (−) and rephosphorylated (+) human HSL variants. Purified HSL variants were dephosphorylated by calf intestinal phosphatase and then phosphorylated by PKA and assayed against TO, MOME, CO and pNPB under saturated substrate concentrations (panels A–E). Panel F shows the TO assay saturation curve of PKA phosphorylated wild type HSL. Data represents means±standard error of triplicate assays of an experiment that is representative of three to five independent experiments. *P<0.05; *** P<0.0005, unpaired t test. The maximal turnover rate and S_0.5_ values of the phosphorylated wildtype enzyme was 2.3 U/mg and 0.72 mM, respectively (panel F).

Dephosphorylated protein was phosphorylated by PKA and assayed for activity directly afterwards. The activities of the wild type, SVA-SS, SVE-SS and AVA-SS variants increased by 87%, 91%, 62% and 58 %, respectively, against triolein and, by 22%, 44%, 41% and 37%, respectively, against the diolein analogue MOME. The activity against CO was increased by 15%, 19% and 21%, respectively for the wild type, SVA-SS and SVE-SS variants. PKA phosphorylation of the AVA-AA mutant had no effect on its activity towards any of the tested substrates (**Figure 8, A–E**). Because the degree of activation in absolute terms varied from experiment to experiment, the results shown in Figure 8 are representative of three to five individual experiments for each assay. In all experiments the activity of the wild type, SVA-SS, SVE-SS and AVA-SS variants increased against TO and MOME and for the wildtype, SVA-SS and SVE-SS variants the activity also increased against CO. In all experiments the activation ranged between 50% and 110% for TO, 20% and 45% for MOME for the four variants and between 5% and 20% for CO for the wild type, SVA-SS and SVE-SS variants (**Figure 8, A–E**). In no experiment was an activation found against the pNPB substrate.

### Kinetic behavior of the PKA-activated wild type enzyme

The kinetic behaviour of the PKA-phosphorylated form of the wild type enzyme when assayed against increasing concentrations of TO is shown in Fig. 8F. The S_0.5_ value was 0.73±0.13 mM and the maximum turnover rate was 2.3±0.11 U/mg (**Figure 8F** **and** **Table 2**). Thus, phosphorylation results in an increase in both turnover and substrate binding with regard to the TO substrate.

## Discussion

In this study we used the baculovirus/insect cell system to generate recombinant human HSL in order to identify the sites phosphorylated by PKA *in vitro* and to study their importance for the enzymatic properties of human HSL. The specific activity of 23 U/mg for the wild type enzyme against a diolein analogue is well in agreement with that previously reported for His-tagged human HSL [25]. Kinetic measurements revealed a maximal turnover rate of 60 U/mg and a S_0.5_ value of 0.15 mM under our pNPB assay conditions. These values are different from those previously published for the human His-tagged enzyme: 1 mM for K_0.5_ and 134 U/mg for k_cat_ (k_cat_ was calculated from the data presented for pNPB activity in [26]). The reason for this apparent discrepancy is most likely found in the assay conditions employed. The solubility limit of pNPB is 1 mM in the absence of mechanical stirring. In the cited work the authors used mechanical stirring, which we did not, as a way of increasing the solubility of pNPB, thus increasing both S_0.5_. and maximal turnover rate.

Mutating Ser554 into either Ala or Glu had little or no effect on the activity of HSL. Even mutating both Ser552 and Ser554 to Ala did not alter activity significantly. This is in agreement with a previous study performed by our group in which mutation of the corresponding sites in rat HSL (Ser563 and Ser565) was found to have no effect on the activity [9], and also with another study on rat HSL reporting that deletion mutants lacking both Ser563 and Ser565 retained full enzymatic activity [27]. In contrast our result is in disagreement with a reported total loss of activity towards lipid substrates upon mutation of Ser563 to Ala and a partial loss of activity upon mutating Ser565 to Ala in the rat enzyme [11]. This contradiction could be a reflection of differences in activity regulation between the rat and the human enzymes. However it should be pointed out that the data presented in our work are based on purified HSL, whereas the data presented in [9], [11] are based on partially purified enzyme and cell extracts, respectively.

In contrast to the lack of effect of mutating Ser552 and Ser554 in human HSL, mutating Ser649 and Ser650 to Ala severely lowered the activity towards lipid substrates. However the same mutations had a lesser effect on the activity of the enzyme towards pNPB, indicating that Ser649 and Ser650 are more important for the turnover of lipid substrates than for the catalytic reaction as such. Studies of the kinetic behaviour of the two enzyme variants showed that there was no significant difference in the S_0.5_ value towards either the TO substrate or the low molecular weight ester pNPB; in contrast the maximum turnover rates for the AVA-AA mutant were 55% and 33% of those of the wild type enzyme towards pNPB and TO, respectively, in agreement with an effect on turnover rather than substrate binding. The reduced activity could be a reflection of increased rigidity in the AVA-AA mutant, possibly leading to impaired product release despite unaltered substrate binding. This would also fit with the finding that the effect on turnover is significantly lower for pNPB, for which the products are butyrate and para-nitrophenol, than for TO, for which the products are oleate and diolein. The binding of a large oleate molecule by van der Waals' interactions would be expected to be stronger than that of a butyrate.

Differential effects of phosphorylation on lipolytic and non-lipolytic activity have previously been observed in experiments where limited proteolysis of recombinant rat HSL abolished activity against lipid substrates, but had a markedly lower effect on the activity towards pNPB [28]. Our results are also in agreement with studies where HSL was inhibited by an esterase inhibitor in the absence of bile salts acting as interface, suggesting the lack of a lid covering the actual active site [25]. However, our results do not rule out the possibility of a lid controlling how tightly lipids are bound to the enzyme.

By employing ^32^P-labeling during the expression of human HSL in the baculovirus/insect cell system, we showed that HSL purified from Sf9 cells is partly phosphorylated. MS analysis of TiO_2_-enriched tryptic digests of the wild type enzyme revealed phosphopeptides containing Ser649 and Ser650 (not shown). The stoichiometry of the background phosphorylation was not determined. However, the observation that prior dephosphorylation of purified human HSL by calf intestinal phosphatase had no effect on the activity of the wild type enzyme (**Figure 2B**), suggests that the background phosphorylation is quantitatively low. The unexpected discovery that HSL is phosphorylated on biologically relevant sites by insect cells indicates that these cells contain active PKA homologues. However, as indicated by our results, this has no significance for enzymatic activity. In mammalian cells HSL is regulated by several kinases and as a result HSL activity undergoes significant modification, as previously shown for several tissues [12], [29], [30]. Thus, even though the actual *in vivo* stochiometry of HSL phosphorylation has never been established, it is evidently higher in mammalian cells than in insect cells.

Upon *in vitro* phosphorylation by PKA, 0.2 mol phosphate per mol of HSL was incorporated. This level was the same for all variants, except for the AVA-AA mutant lacking Ser649 and Ser650, which only showed trace amounts of incorporated phosphate. MS analysis revealed phosphorylation of several sites. MS/MS analysis of the peptide containing Ser649/Ser650 (RSSQGATQMPLYSSPIVK) suggested that the major part of the phosphorylation of this peptide was present on Ser650 rather than Ser649. This is somewhat in disagreement with our previous work on rat HSL, where phosphopeptide mapping followed by radiosequencing of HSL isolated from isoproterenol-stimulated adipocytes revealed two types of phosphopeptides, a monophosphorylated variant with phosphate on Ser660 and a diphosphorylated peptide with phosphate on both Ser659 and Ser660 [9]. It is possible that species differences account for these apparent discrepancies. However, it should be pointed out that Ser659 in rat HSL (Ser649 in human HSL) is not an optimal PKA consensus site and that previous results from our lab suggested that Ser659 is phosphorylated at a slower rate than Ser660 [9]. *In vivo* evidence for phosphorylation of Ser650 and Ser552 in human HSL has been provided by several studies. Increased levels of cyclic AMP as well as the onset of exercise increase phosphorylation of Ser650 in human skeletal muscle, whereas phosphorylation of Ser552 and Ser554 is unchanged under these conditions [13]. However it was reported in a recent study that both Ser552 and Ser650 were phosphorylated in response to β-adrenergic stimulation in 3T3-L1 adipocytes and after the onset of exercise in skeletal muscle [29]. Such discrepancies could indicate that phosphorylation of Ser650 and Ser552 might be due to the action of different kinases, which is also reflected in our *in vitro* results.

MS analysis showed that the SVA-SS mutant was phosphorylated on Ser552, whereas this was not the case for the SVE-SS mutant, suggesting that the charged Glu554 in this mutant exerts a negative effect on subsequent phosphorylation of Ser552 by PKA. This is expected on the basis of previous work showing that phosphorylation of the site corresponding to Ser554 in human HSL prevents subsequent phosphorylation of the site corresponding to Ser552 by PKA, indicating that phosphorylation of Ser554 plays an antilipolytic role [3], [4]. However, as the SVA-SS, SVE-SS and AVA-SS variants were all activated against TO it seems that any interaction between Ser552 and Ser554 (Ser563 and Ser565 in rat HSL) only has relevance *in vivo*.

Phosphorylation by PKA increased the activity of the wild type human HSL against the triglyceride substrate TO by 87%. This is in line with reported data for both rat and human HSL [8], [31] and the level of activation was similar for all investigated HSL variants, except for the AVA-AA mutant, which showed no activation in response to phosphorylation by PKA. Phosphorylation also increased activity towards the diglyceride substrate MOME by at least 22% for all variants except AVA-AA. We also recorded a slight activation against CO. To our knowledge this is the first time activation towards a diglyceride substrate has been detected *in vitro*. With regard to the activation against CO, studies in which partially purified HSL preparations from adrenal cortex and *corpus luteum* were used have suggested that PKA phosphorylation increases the activity towards cholesteryl ester substrates [32], [33]. Analysis of the kinetic behavior of the wild type enzyme against TO revealed that not only did phosphorylation increase maximum turnover rate, but it also increased S_0.5_ compared to the non-phosphorylated enzyme. This is logical in the view of the known translocation of HSL to the lipid droplet after stimulation of lipolysis. Once HSL is placed in the hydrophobic milieu in the droplet it will be in an environment saturated with substrate and activity will not benefit from a strong substrate interaction, but rather from an increased release rate of product molecules. Considering that the S_0.5_.values of AVA-AA for pNPB and TO is similar to those of the non-phosphorylated wild type enzyme, it seems likely that the conformational changes induced by the phosphorylation event affect other structural features than those affected by the mutation of Ser649 and Ser650 to Ala. An explanation for this phenomenon is not obvious and underlines the need for a crystal structure of HSL. It could be speculated that the HSL molecule following phosphorylation by PKA adopts a more open and flexible conformation rendering the enzyme surface more hydrophobic enabling a freer access to the lipid binding site in return for weaker binding, which in turn is not rate limiting inside the lipid saturated environment of a lipid droplet.

Thus *in vitro* activation of HSL might reflect a fine-tuning of the enzyme for the lipid saturated environment of lipid droplets and is mediated solely by phosphorylation of Ser649 and Ser650 by PKA. In contrast, activation of HSL *in vivo* relies on the phosphorylation of several sites, and is a result of a complex interplay between HSL, PKA, perilipin and most likely other key players, such as phosphatases, additional kinases and lipid droplet associated proteins, leading to a more than 50-fold increase in the lipolytic rate. Previous work on rat HSL has shown that Ser565, Ser659 and Ser660 (corresponding to Ser554, Ser649 and Ser650 in human HSL) are all determinants of the translocation event [10], whereas the role of Ser563 remains elusive.

In conclusion, we have demonstrated that activation of human HSL against lipid substrates in response to phosphorylation by PKA *in vitro* is governed by Ser649 and Ser650 and that this activation involves alterations in the kinetic behavior of the enzyme with increases in both maximum turnover rate and S_0.5_. Moreover, mutation of Ser649 and Ser650 results in loss of lipolytic activity, with lesser effect on non-lipolytic activity. Taken together, this suggests that Ser649 and Ser650 are located in the vicinity of a lipid binding region and that phosphorylation by PKA controls the accessibility of this region.
